# Inhibition of Glycolysis Reduces Disease Severity in an Autoimmune Model of Rheumatoid Arthritis

**DOI:** 10.3389/fimmu.2018.01973

**Published:** 2018-09-03

**Authors:** Georges Abboud, Seung-Chul Choi, Nathalie Kanda, Leilani Zeumer-Spataro, Derry C. Roopenian, Laurence Morel

**Affiliations:** ^1^Immunology, and Laboratory Medicine, Department of Pathology, University of Florida, Gainesville, FL, United States; ^2^The Jackson Laboratory, Bar Harbor, ME, United States

**Keywords:** arthritis, mouse, follicular helper T cells, glycolysis, metabolism

## Abstract

The K/BxN mouse is a spontaneous model of arthritis driven by T cell receptor transgenic CD4^+^ T cells from the KRN strain that are activated by glucose-6-phosphate isomerase (GPI) peptides presented by the H-2^g7^ allele from the NOD strain. It is a model of autoimmune seropositive arthritis because the production of anti-GPI IgG is necessary and sufficient for joint pathology. The production of high levels of anti-GPI IgG requires on the expansion of CD4^+^ follicular helper T (Tfh) cells. The metabolic requirements of this expansion have never been characterized. Based on the therapeutic effects of the combination of metformin and 2-deoxyglucose (2DG) in lupus models that normalized the expansion of effector CD4^+^ T cells. We showed that the CD4^+^ T cells and to a lesser extent, the B cells from K/BxN mice are more metabolically active than the KRN controls. Accordingly, preventive inhibition of glycolysis with 2DG significantly reduced joint inflammation and the activation of both adaptive and innate immune cells, as well as the production of pathogenic autoantibodies. However, contrary to the lupus-prone mice, the addition of metformin had little beneficial effect, suggesting that glycolysis is the major driver of immune activation in this model. We propose that K/BxN mice are another model in which autoreactive Tfh cells are highly glycolytic and that their function can be limited by inhibiting glucose metabolism.

## Introduction

The concept that cellular metabolism controls the functions of immune cells has greatly evolved in the past few years, with T cells leading the way over other immune cell types (1, 2). Quiescent T cells produce ATP by oxidizing glucose, fatty acids or glutamine. TCR/CD28 stimulation upregulates glucose uptake to generate ATP as well as biosynthesis intermediates through the reduction of pyruvate into lactate in a process known as aerobic glycolysis. A number of metabolic inhibitors block key nodes of T cell metabolism. 2-deoxyglusose (2DG) inhibits glucose metabolism at its first enzymatic steps. Metformin reduces mitochondrial respiration in activated T cells (3), which is consistent with its inhibition of the electron transport chain complex 1 (4). Based on the drastic differences in metabolic requirements and the critical role of effector T cells in autoimmune diseases, T cell metabolism has been proposed as a target for immunotherapy (5, 6). We and others have shown the existence of multiple immune metabolism defects in lupus patients and mouse models of the disease (7). We have also shown that the combination of 2DG and metformin normalized the metabolism of lupus CD4^+^ T cells and reverse disease in multiple mouse models (3, 8).

Activated CD4^+^ T cells in rheumatoid arthritis (RA) patients shunt glucose flux to the pentose phosphate pathway leading to a hyper-reduced state (9), due in part in a deficiency in phosphofructokinase PFKFB3 (10). Treatments with drugs increasing mitochondrial oxidation (9) or preventing FA synthesis (11) normalized the metabolism of these T cells, and decreased their infiltration into synovial tissue implanted in humanized mice. In the zymosan-induced model of arthritis, treatment with fructose 1,6-bisphosphate (FBP), the product of PFKFB3, attenuated disease severity (12). Lymphocytes infiltrating the joints of RA patients express a high levels of hexokinase *HK2*, indicating that they are also highly glycolytic, which is consistent with their activation status (13). Inhibition of glycolysis with bromo-pyruvate greatly reduced disease severity in the SGK mouse, another zymosan-induced model of arthritis driven by Th17 cell expansion (13), which is consistent with Th17 cell dependence on glycolysis (14). Fibroblast-like synoviocytes (FLS) are also highly glycolytic in the inflamed joints of RA patients and in the murine K/BxN (KBN) serum transfer model (15). In this model of immune complex induced joint inflammation, bromo-pyruvate prevented and reversed disease, suggesting that FLS are the primary target (15). Overall, there is evidence of metabolic disturbances in the immune and joint cells associated with RA, with glucose metabolism playing a key role.

The KBN mouse is a spontaneous model of arthritis driven by T cell receptor transgenic CD4^+^ T cells from the KRN strain that are activated by glucose-6-phosphate isomerase (GPI) peptides presented by the H-2^g7^ allele from the NOD strain (16). It is a model of autoimmune arthritis because the production of anti-GPI IgG is necessary and sufficient for joint pathology. Indeed KBN serum induces disease by activating inflammatory innate cells, especially neutrophils, in the effector phase of the disease leading to joint damage (17). GPI is highly expressed in the arthritic joint (18), and the formation of anti-GPI immune complexes triggers an inflammatory cascade leading to tissue damage. Based on therapeutic effects of the combination of metformin and 2DG in lupus mouse models that normalized the expansion of effector CD4^+^ T cells (3, 8), we hypothesized that these metabolic inhibitors would also have a beneficial effect in autoimmune arthritis. We showed that the CD4^+^ T cells and to a lesser extent, the B cells from KBN mice are more metabolically active than the KRN controls. Accordingly, inhibition of glycolysis with 2DG significantly reduced joint inflammation and the activation of both adaptive and innate immune cells, as well as the production of pathogenic autoantibodies. However, contrary to the lupus-prone mice, the addition of metformin had little beneficial effect, suggesting that glycolysis is the major driver of immune activation in this model.

## Materials and methods

### Mice and disease assessment

KBN mice were F1 hybrids between TCR transgenic (Tg) KRN females and NOD males, both obtained from The Jackson Laboratory. The KRN strain was maintained by out-crossing to C57BL/6 (B6), and the presence of the Tg was detected by PCR (19). B6 mice were also used as controls in some experiments. Both males and females were used. Treatment with 2DG (6 mg/ml) with or without metformin (3 mg/ml) was administered in drinking water as previously described (3), starting between 30 and 35 days of age for the entire duration of the experiment. Littermate controls received plain water. Serum transfers were conducted as described (19). Briefly, 300 ul of pooled serum collected from metformin + 2DG (Met+2DG), 2DG-treated or control mice was injected at day 1 and 3 into 5–6 month old KRN males. Joint thickness was measured on all 4 limbs 3 times a week with a caliper and disease severity was assessed on a semi-quantitative scale as previously described (19). Results are reported as the sum measurements or scores for all 4 limbs. Three cohorts of 2DG treatment and controls were performed, as well as three additional cohorts of metformin + 2DG (Met+2DG), 2DG alone, and controls. Two cohorts of serum transfers were performed with sera from 2DG-treated and control mice, as well as with sera from Met+2DG, 2DG-treated, or control mice. Results were presented on pooling cohorts. The levels of serum anti-GPI IgG were measured by ELISA with in plates coated with 5 μg/ml recombinant GPI (19) with serum samples diluted 1:1000. This study was carried out in strict accordance with the recommendations in the Guide for the Care and Use of Laboratory Animals of the animal Welfare Act and the National Institutes of Health guidelines for the care and use of animals in the biomedical research. All animal protocols were approved by the Institutional Animal Care and Use Committee (IACUC) of the University of Florida, Gainesville (OLAW Assurance # A3377-01).

### Flow cytometry

Single-cell suspensions were prepared from spleens and brachial lymph nodes using standard procedures. After red blood cell lysis, cells were blocked with anti-CD16/32 Ab (2.4G2), and stained in FACS staining buffer (2.5% FBS, 0.05% sodium azide in PBS). Fluorochrome-conjugated Abs used were to B220 (RA3-6B2), BCL6 (K112-91), CD3 (145-2C11), CD4 (RAM4-5), CD8 (53-6.7), CD11b (M1/70), CD11c (HL3), CD25 (PC61.5), CD44 (IM7), CD62L (MEL-14), CD69 (H1.2F3), CD95 (Jo2), CD98 (RL388), CD138 (281-2), CD162 (PSLG-1, 2PH1), CD279 (PD-1, RMP1-30), Foxp3 (FJK-16s), I-A/I-E (MHC-II, M5/114.15.2), IL-17A (TC11-18h10.1), Ly6C (HK1.4) and Ly6G (1A8), Ly-77 (GL7), pAKT-s473(SDRNR), pE4-BP (236B4), pS6 (D57.2.2E), and PDCA-1 (129C1), purchased from BD Biosciences, Thermo Fisher, BioLegend, or Cell Signaling Technology. Monocyte/macrophages were gated as CD3– CD11b+Ly6Chi while neutrophils were CD3– CD11bhi Ly6G+. Follicular helper T cells were stained as previously described (3) in a three-step process using purified CXCR5 (2G8) followed by biotinylated anti-rat IgG (Jackson ImmunoResearch Laboratory) then PerCP-labeled streptavidin in FACS staining buffer on ice. For the intracellular staining, cells were fixed and permeabilized with FOXP3 stating buffer (Thermo Fisher) according to the manufacturer's protocol.

### Metabolic measurements

Single splenocyte suspensions were enriched for total CD4^+^ T cells or B cells by negative selection with magnetic beads (Miltenyi). Glycolysis recorded as Extracellular Acidification Rate (ECAR) and Oxygen Consumption Rate (OCR) were measured using a XF96 Extracellular Flux Analyzer (Agilent Seahorse) in a standard mitochondrial stress assay with non-buffered RPMI medium (Sigma) supplemented with 2.5 uM dextrose, 2 mM glutamine and 1 uM Sodium Pyruvate. Samples were assayed at least in triplicates for 3 successive 8 min time intervals between inhibitor injections. Baseline ECAR and OCR values were averaged between replicates for these 3 time points. Mitochondrial spare respiratory capacity (SRC) was defined as the OCR difference between baseline and maximum respiration after injection of Carbonyl cyanide 4-(trifluoromethoxy) phenylhydrazone (FCCP).

## Statistical analysis

Analyses were performed using GraphPad Prism 6.0. Unless indicated, graphs show means and standard errors of the mean. Comparisons were performed using two-tailed unpaired student *t*-tests and 2-way ANOVA. Statistical significance was defined as *P* < 0.05.

## Results

### KBN lymphocytes have a high cellular metabolism

**Figure 1:**
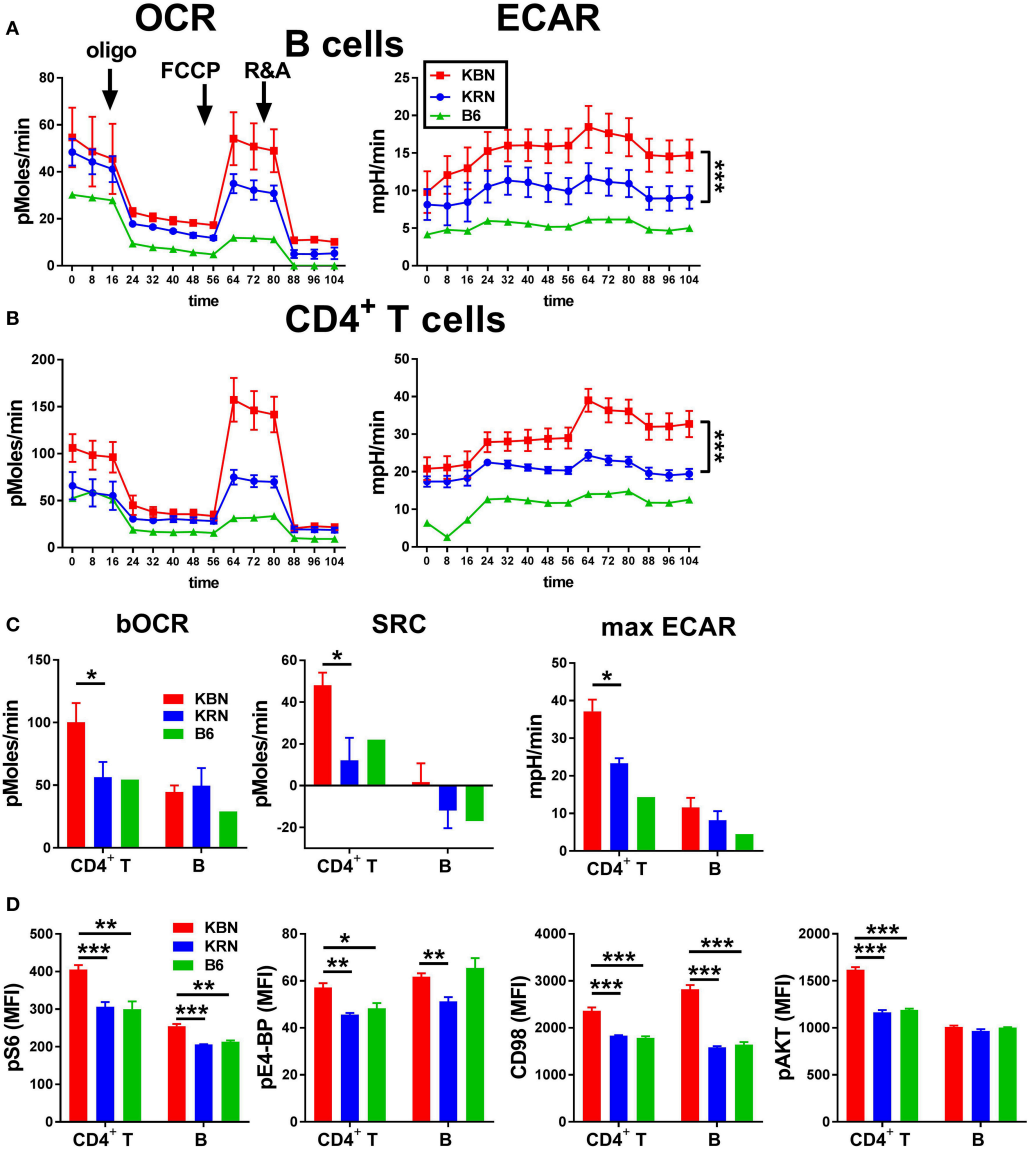
KBN lymphocytes have a high metabolism. Mitochondrial stress test in B cells **(A)** and CD4^+^ T cells **(B)** purified from 6 week old KRN and KBN females (*N* = 3) with one B6 female shown as control. ECAR plots were compared between KRN and KBN mice by 2-way ANOVA. **(C)**. Basal OCR and SRC as well as maximum ECAR in CD4^+^ T cells and B cells calculated from data shown in **(A,B)**. **(D)**. pS6, pE4-BP, CD98 and pAKT in CD4^+^ T cells and B cells (*N* = 5). *t*-tests were used in C and D. *p < 0.05; **p < 0.01; ***p < 0.001.

We compared the basis metabolic parameters of total B cells and CD4^+^ T cells between KBN and KRN mice, which share the same Tg TCR repertoire, at 6 weeks of age, which is an early disease stage for KBN mice. A B6 mouse was used as control. KBN B cells showed a similar OCR profile as KRN, but glycolysis was globally higher in KBN than KRN B cells, especially when mitochondrial ATP production was inhibited after oligomycin and FCCP treatment (Figure 1A). KBN CD4^+^ T cells showed a higher basal OCR and a higher SRC, as well as a higher glycolysis, especially, as for B cells, when mitochondrial ATP production was inhibited (Figures 1B,C). Consistent with a higher metabolism, KBN CD4^+^ T cells and B cells also showed a higher mTORC1 activation as measured by pS6, pE4-BP and CD98 expression, as compared to KRN (Figure 1D). Overall, these results show that consistent with their autoantibody production for B cells (20) and their enhance effector functions for CD4^+^ T cells (1), KBN lymphocytes are more metabolically active than KRN controls.

### Inhibition of glucose metabolism inhibits the development of joint inflammation

**Figure 2:**
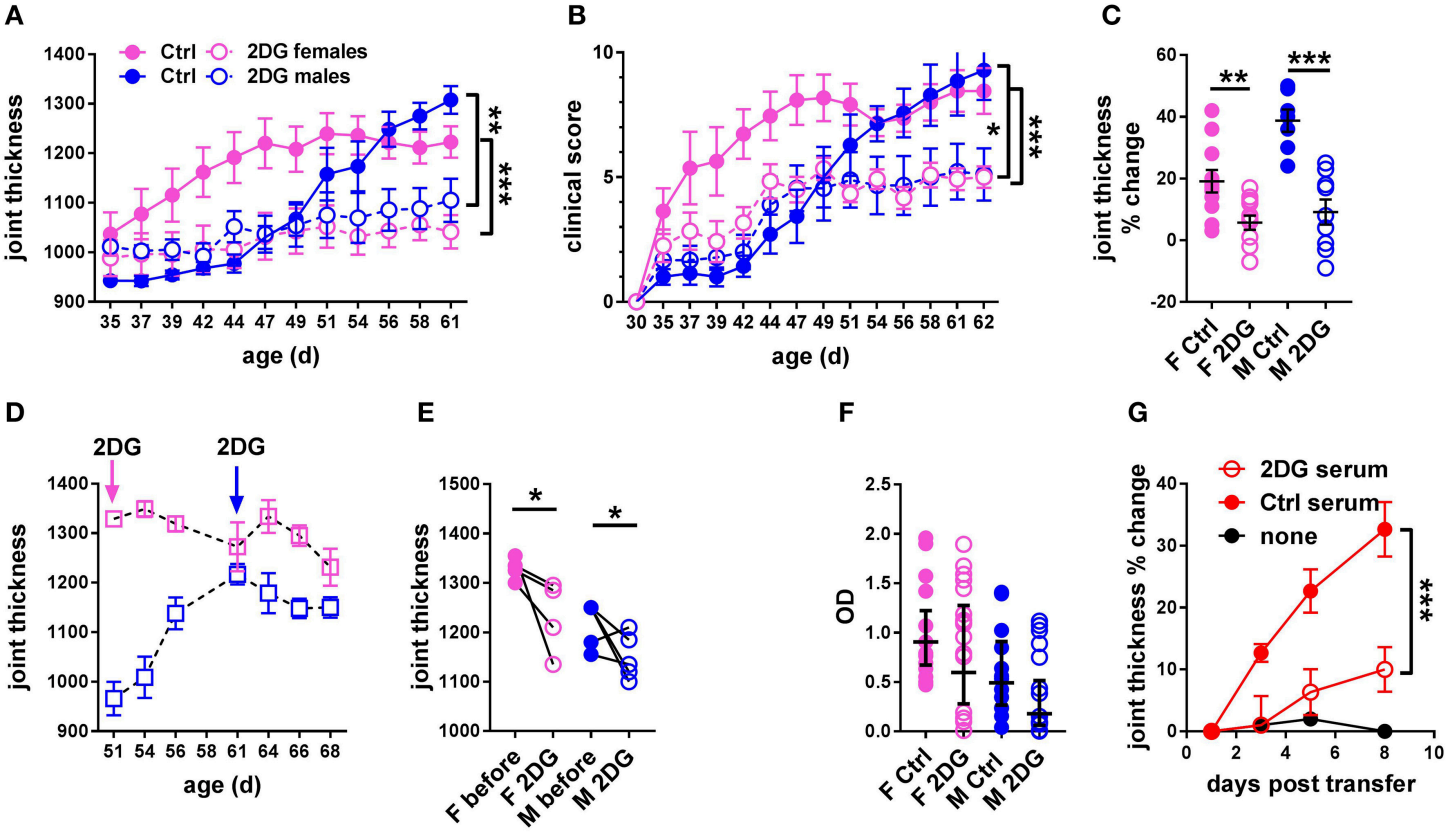
Glycolysis inhibition reduced joint inflammation in KBN mice. Time course of joint thickness **(A)** and clinical scores **(B)** in mice treated or not with 2DG (female controls *N* = 11; female 2DG *N* = 12; male controls *N* = 7; male 2DG *N* = 9). **(C)**. Percent change in joint thickness between d 35 and d 61 in these mice (*t*-tests). **(D)**. Time course of joint thickness in mice in which treatment with 2DG started after severe joint inflammation was established, at 51 d old for females and 61 d old for males (as indicated by arrows). **(E)**. Changes in joint thickness after 2DG treatment shown in **(D)** in individual mice (initial and terminal measurements, paired *t*-tests). **(F)**. Serum anti-GPI IgG in mice treated preventively or not with 2DG as shown in **(A,B)** (geometric means ± 95% confidence intervals). **(G)**. Joint thickness in KRN mice after transfer of serum from KBN mice treated with 2DG or controls (N = 6 each). An uninjected KRN mouse is included as control. Plots in **(A,B,G)** were compared by 2-way ANOVA. *p < 0.05; **p < 0.01; ***p < 0.001.

We first addressed whether treatment with 2DG initiated prior to disease onset. Robust joint swelling developed in KBN females with earlier onset than males (Figure 2A). Consequently, the results are reported separately for males and females. Treatment with 2DG starting at 5 weeks of age when joint inflammation was minimal significantly decreased swelling (Figure 2A), as well as clinical scores (Figure 2B). There was also a significant effect of the treatment as measured by the individual changes in joint thickness after about 2 months of continued treatment (Figure 2C). Thus, 2DG inhibited but did not fully abrogate the development of the clinical features of KBN arthritis. We then addressed whether 2DG treatment affected joint inflammation in mice in which treatment was initiated when disease was established. 2DG therapy initiated 51 days (females) and 61 days caused partial reduction of joint inflammation (Figures 2D,E). However, levels of serum anti-GPI IgG were not decreased in the majority of mice receiving the preventive 2DG treatment (Figure 2F) but sera from 2DG-treated mice induced less inflammation as compared to control sera when transferred into KRN mice (Figure 2G). This indicates that glucose inhibition induced qualitative differences in anti-GPI IgG arthrogenic activity.

### Inhibition of glucose metabolism affects multiple cell populations involved in KBN arthritis

**Figure 3:**
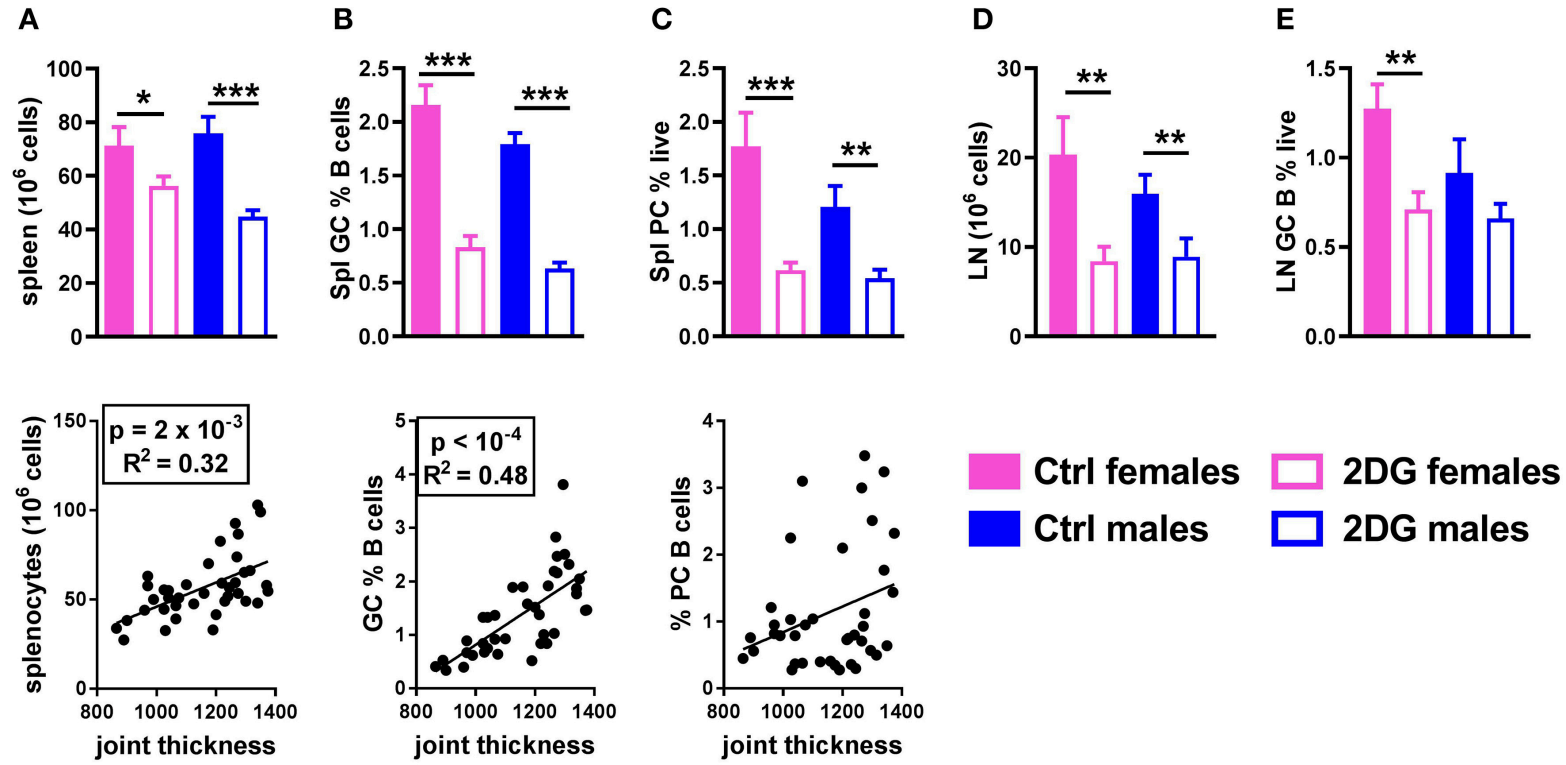
Glycolysis inhibition reduced lymphoid expansion as well as GC B cells and plasma cell differentiation. Mice treated with 2DG showed a reduced number of splenocytes **(A)** and frequency of GL7^+^CD95^+^ GC B cells **(B)**, both of which were correlated with disease severity, as well as a reduction of the frequency of CD138^+^B220^lo/neg^ plasma cells, which was not correlated with disease activity **(C)**. 2DG also reduced the number of cells **(D)** and the frequency of GC B cells **(E)** in the JDLN. Mean values between the treated and control mice were evaluated with *t*-tests. *p < 0.05; **p < 0.01; ***p < 0.001. Correlations between joint thickness and immune variables were computed by pooling the 4 groups of mice and evaluated with a Pearson test as indicated for each graph.

The preventive 2DG treatment reduced lymphoid expansion in the spleen and the number of splenocytes correlated with disease severity (Figure 3A). The total percentage of B cells and CD4^+^ T cells was unchanged (data not shown). 2DG reduced the frequency of germinal center (GC) B cells, which also correlated with disease activity (Figure 3B), and the frequency of splenic plasma cells (Figure 3C). The lack of correlation between this latter variable and joint swelling may indicate that the majority of plasma cells or plasmablasts have moved to the bone marrow. The number of cells (Figure 3D) and the frequency of GC B cells (Figure 3E) were also reduced in the joint draining lymph node (JDLN).

**Figure 4:**
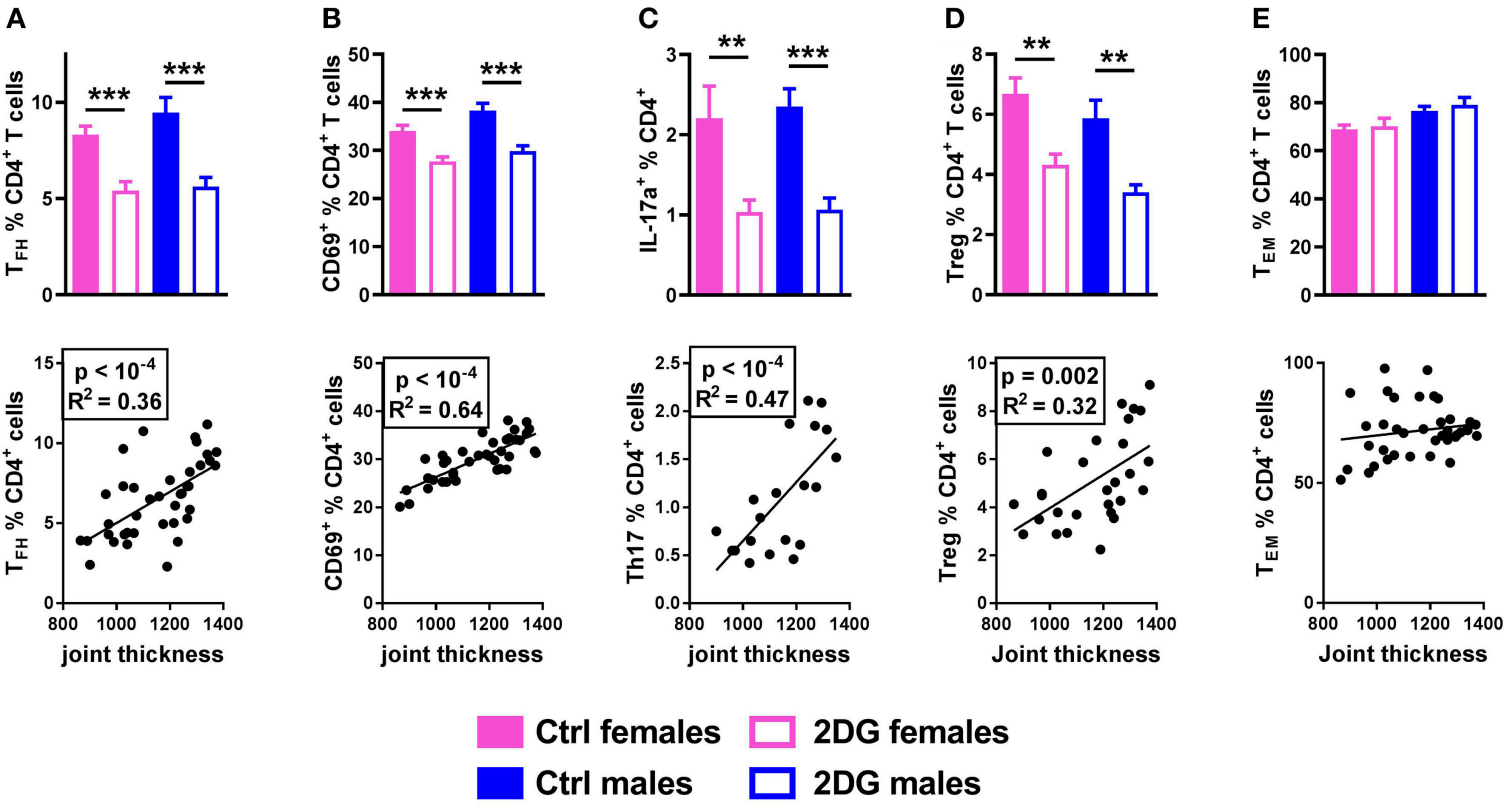
Glycolysis inhibition reduced Tfh and Th17 cell expansion. Mice treated with 2DG showed a reduced frequency of Tfh **(A)**, CD4^+^ T cells expressing the activation marker CD69 **(B)**, Th17 **(C)** and Treg **(D)** cells, all of which were correlated with disease severity. The frequency of T_EM_ cells was not decreased by the 2DG treatment and T_EM_ frequency was no correlated to disease severity **(E)**. Mean values between 2DG-treated and control mice were evaluated with *t*-tests. Correlations were evaluated with the Pearson test. **p < 0.01; ***p < 0.001.

The frequencies of Tfh cells (Figure 4A), activated CD69^+^CD4^+^ T cells (Figure 4B) and IL-17A producing CD4^+^ T cells (Figure 4C) were greatly reduced by the 2DG treatment, all of which correlated with disease severity. 2DG also decreased the frequency of Foxp3^+^CD25^+^CD4^+^ regulatory T cells (Treg), a subset that has not reported to be involved in this model, and the frequency of Tregs was also correlated with disease severity (Figure 4D). As we have previously reported in lupus mice (8), 2DG alone did not reduced the frequency of CD44^+^CD62^−^CD4^+^ effector memory T cells (T_EM_), and the frequency of these cells did not correlate with joint inflammation (Figure 4E).

**Figure 5:**
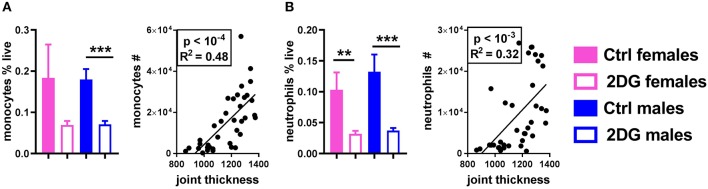
Glycolysis inhibition reduced the frequency of neutrophils and monocytes in the JDLN and altered their activation. The frequency of monocytes **(A)** and neutrophils **(B)** was reduced by 2DG treatment, and the number of these cells in the joint draining lymph node correlated with disease severity. Mean values between the treated and control mice were evaluated with *t*-tests. Correlations were evaluated with the Pearson test. **p < 0.01; ***p < 0.001.

Finally, class-switched autoantibodies resulting from the expansion of GC B cells and Tfh cells require myeloid cells to induce inflammation in the joint (21). 2DG treatment reduced the number of monocytes (Figure 5A) and neutrophils (Figure 5B) in the JDLN. A positive correlation was observed between the numbers of these myeloid cells and joint inflammation (Figure 5). Overall, the disease attenuation that occurred in KBN mice treated with 2DG correlated with a reduction of multiple immune cells whose expansion has been reported to contribute to autoimmune pathogenesis in this model: GC B cells, Tfh and Th17 CD4^+^ T cells and myeloid cells.

### The addition of metformin does not improve the effect of 2DG treatment

**Figure 6:**
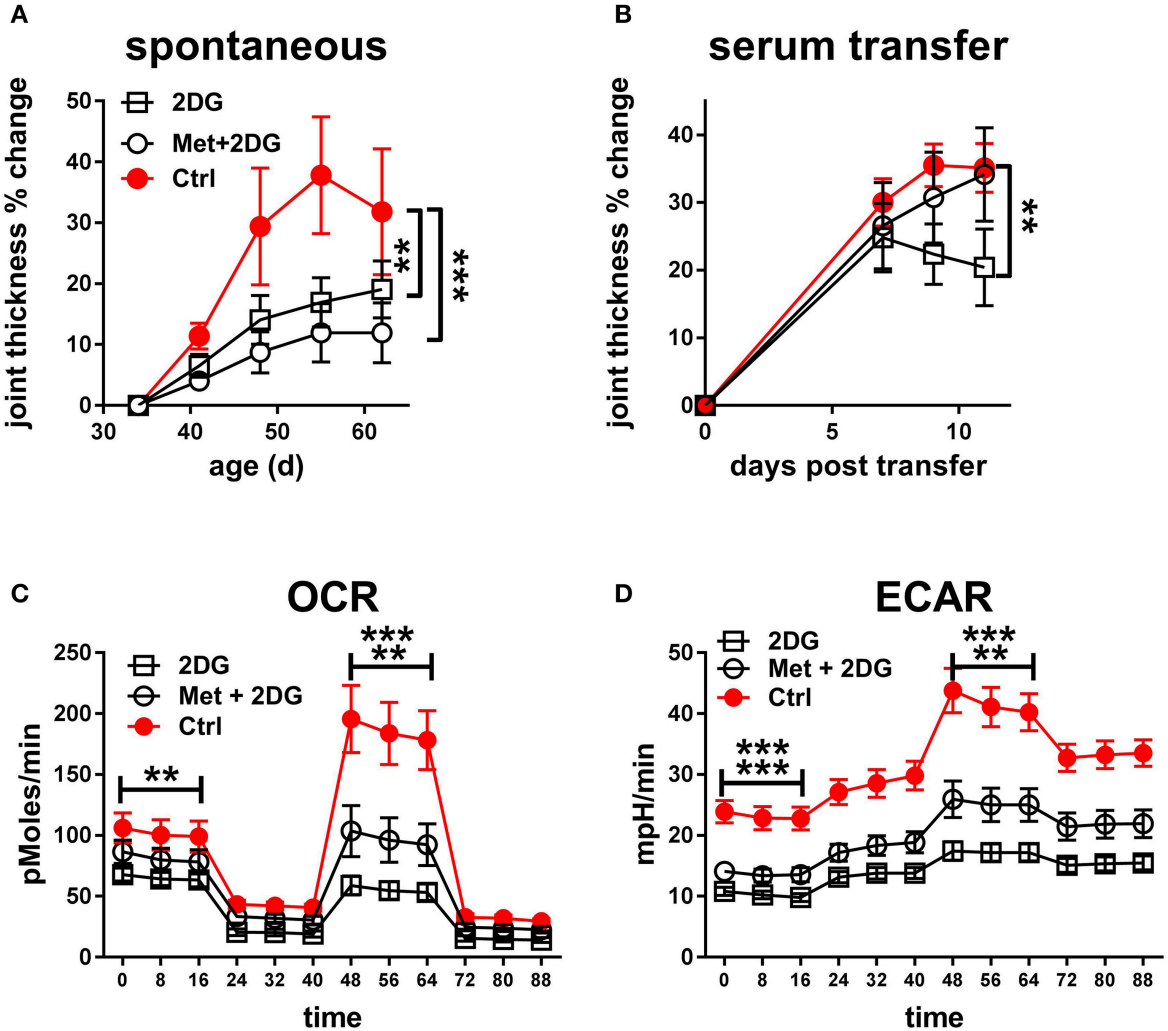
Metformin does not improve the protective effect of 2DG: **(A)**. Time course of joint thickness in KBN mice treated with Met+2DG (8 females and 7 males), 2DG (9 females and 6 males) or untreated controls (5 females and 2 males). The 2DG treated and control mice were contemporaneous to the Met+2DG treated mice, and were a different cohort from the mice presented in Figure 2. The results were pooled between males and females. Treatments were compared to controls by a 2-way ANOVA. **(B)**. Joint thickness in KRN mice after transfer of serum from KBN mice treated with Met+2DG, 2DG, or controls (*N* = 8 recipient each). Mean terminal values between treated and control mice were evaluated with *t*-tests. For both A and B, results were expressed as percentage change from the first day of treatment or serum transfer. OCR **(C)** and ECAR **(D)** from a mitochondrial stress test in CD4^+^ T cells purified from the KBN mice at the end of the treatment with Met+2DG, 2DG or control. Statistical analyses shown for the mean values of basal ECOR and ECAR (left of the graphs) and SRC or maximal ECAR (right of the graphs) between the treated and control mice evaluated with *t*-tests. The first row indicates comparison between 2DG and control, and the second row between 2DG and controls. **p < 0.01; ***p < 0.001.

Since the 2DG treatment did not completely prevent disease in KBN mice (Figure 2), we tested whether acted synergistically protection as we have documented in lupus-prone mice (3). The combined treatment of metformin and 2DG (Met+2DG) did not significantly change clinical outcomes as compared to mice that were treated with 2DG alone (Figure 6A). However, sera obtained from the Met+2DG-treated mice induced significantly more joint inflammation than that of 2DG-treated mice when transferred into KRN mice (Figure 6B).

**Figure 7:**
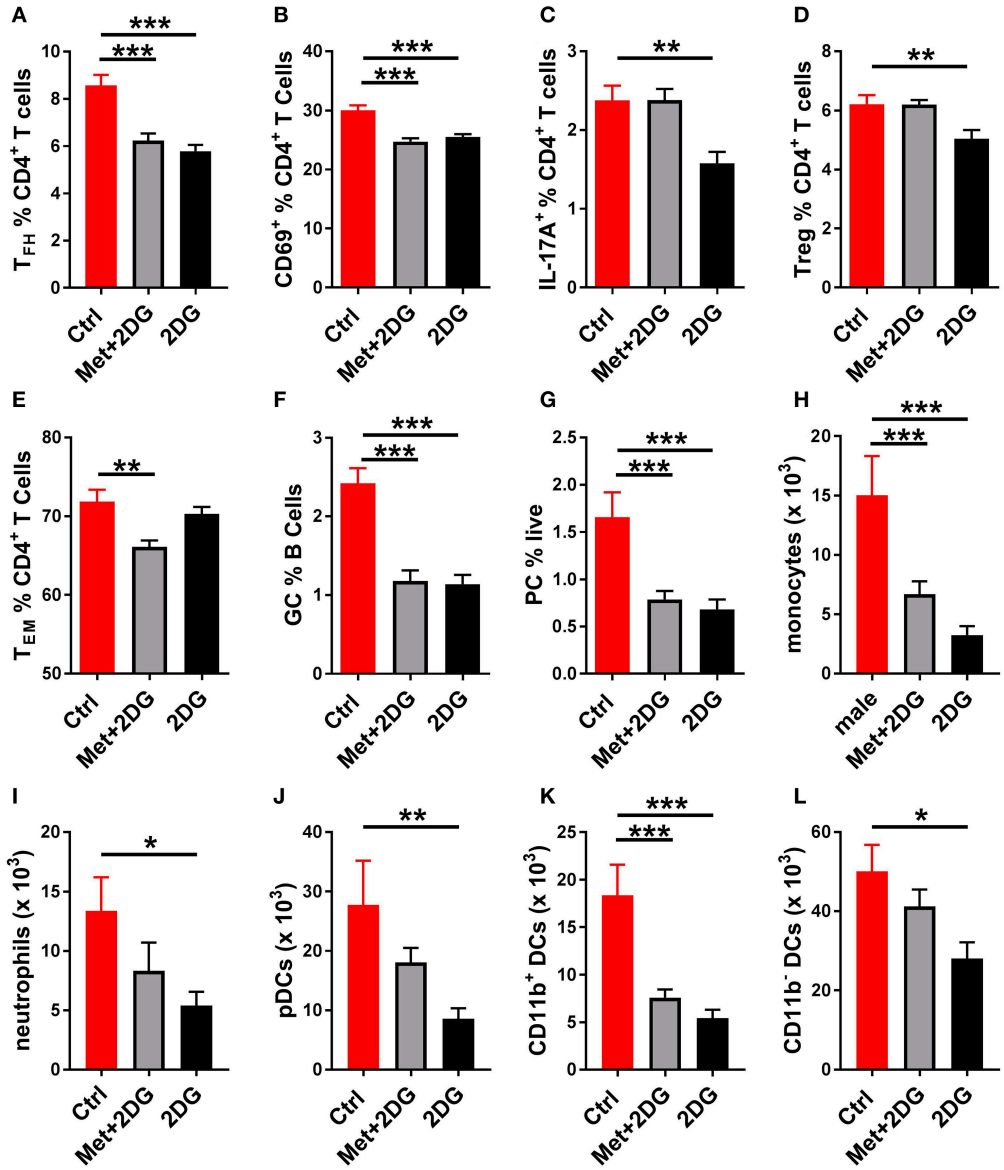
Differential effects of Met+2Dg and 2DG alone *in vivo* treatment on the expansion of immune subsets. Frequency of Tfh cells **(A)**, CD4^+^ T cells expressing the activation marker CD69 **(B)**, IL-17A producing CD4^+^ T cells **(C)**, Treg cells **(B)**, Tem cells **(E)**, GC B cells **(F)**, and plasma cells **(D)** in the spleen. Numbers of monocytes **(H)**, neutrophils **(I)**, pDCs **(J)**, CD11b^+^ **(K)** and CD11^−^ **(L)** in the DCs JDLN. There was not significant difference between males and females, and their samples were pooled. Mean values between treated and control mice were evaluated with one-way ANAOVA and multiple comparison tests. *p < 0.05, **p < 0.01; ***p < 0.001.

To understand the basis for these differences, we compared the effect of the two treatments on the metabolic parameters of CD4^+^ T cells of these mice. 2DG significantly decreased both respiration (Figure 6C) and glycolysis (Figure 6D), as measured at either basal or maximal levels, but the addition of metformin did not improve this metabolic inhibition. Further, the addition of metformin did not improve the inhibitory effect of 2DG on the expansion of Tfh cells, CD69^+^CD4^+^ T cells, GC B cells and plasma cells (Figures 7A,B,E,F). Interestingly, the addition of metformin eliminated the inhibitory effect of 2DG on Th17 and Treg cell expansion (Figures 7C,D). On the contrary, Met+2DG decreased the frequency of Tem while the effect of 2DG alone was not significant (Figure 7G), confirming the results obtained with the previous cohort (Figure 4E). Finally, the combination of Met+2DG had a similar effect on monocytes and CD11b^+^ myeloid dendritic cells than 2DG alone (Figures 7H,K). However, the addition of metformin negated the beneficial effect of 2DG on neutrophils, pDCs and CD11b^negative^ DCs (Figures 7I,J,L). Overall these results suggest that glycolysis is the driving metabolic process in the KBN model.

## Discussion

To produce high levels of anti-GPI IgG, KBN arthritis strongly relies on the expansion of CD4^+^ follicular helper T (Tfh) cells and IL-21 they secrete, both which are required for the production of high-affinity class-switched antibodies (22). Approximately 2/3 of RA patients (“seropositive patients”) produce class-switched rheumatoid factor and/or anti-cyclic citrullinated peptide (CCP) autoantibodies. The therapeutic efficacy of depleting B cells with rituximab and blocking CD4^+^ T cell co-stimulation with abatacept (CTLA4-Ig) strongly support a strong contribution of B and T cells leading to the production of autoantibodies to the pathogenesis of RA (23). Furthermore, a population related to Tfh cells coined peripheral helper T cells (Tfp) is expanded in the serum and the joints of seropositive RA patients (24), supporting a critical role of such T cells in providing help to autoantibody producing B cells in RA. Finally, the expansion of a novel population of “Tfh-like” CXCR5^+^ Th17 cells correlates with disease activity in RA patients, and it is refractory to TNF blockade (25). Tfh17 cells are also expanded in KBN mice under the control of the microbiome (26). Recent studies have shown that Th17 cells are dispensable for the development of disease (27) and that the gut microbiome regulates KBN Tfh but not Th17 cells (28). Moreover, the analysis of K/BxN IL-21-VFP.IL-17A-GFP mice directly showed a robust expansion of IL-21-producing CD4^+^ T cells but very few IL-17-procuding CD4^+^ T cells (Roopenian, unpublished). However, transfer of *Bifidobacterium adolescentis*, a bacterium that drives human Th17 cell differentiation, exacerbated disease in the same KBN model (29). Regardless of whether the expansion of Th17 cells is pathogenic in the KBN model or the result of a bystander effect, it corresponds to the well-documented expansion of Th17 cells in RA patients (30–35).

In this study, we showed that CD4^+^ T cells and, to a lesser extent, B cells from KBN mice are highly metabolic, with high levels of mTORC1 activation, as well as mTORC2 in CD4^+^ T cells. Early continued treatment with an inhibitor of glycolysis significantly decreased the severity of joint inflammation. A modest therapeutic effect was also when mice with established disease, most likely due to the rapid onset of this severe disorder. The preventive efficacy of 2DG was associated with a decreased frequency and numbers of cell subsets that have been associated with disease in this model. We hypothesize that the enhanced metabolism of B cells in the KBN model is secondary to their stimulation by highly metabolic autoreactive CD4^+^ T cells, and that 2DG affects these activated B cells indirectly through a primary effect on Tfh cells. In spontaneous mouse models of lupus, we have shown that the inhibition of glucose metabolism selectively targets autoreactive Tfh cells (Choi et al. unpublished). Moreover, mTORC1 activation has been linked to autoreactive Tfh cell expansion by promoting the translation of Bcl6, the master regulator of Tfh cell gene expression, in the lupus -like *Def6*^*tr*/*tr*^*Swap70*^−/−^ DKO mouse model (36). Th17 cells are known to be highly glycolytic (14), including in the context of arthritis (13). We propose that KBN mice are another model in which autoreactive Tfh cells are highly glycolytic and that their function can be limited by inhibiting glucose metabolism. Icos signaling activates mTOR in Tfh cells, which in turn drives glycolysis (37). A recently completed clinical trial showed that blocking mTOR activation with rapamycin in lupus patients reduced disease activity, including arthritis (38). These results, combined with the results presented in this study with KBN mice suggest that treatments with rapamycin may also be beneficial in seropositive RA patients.

Although we only assessed a relative short-term treatment with 2DG, joint inflammation was not reduced to pre-disease status, therefore suggesting that some pathogenic effector functions were glucose-independent. It is possible that the dosage we used was insufficient. Arguing against this possibility is the profound reduction of CD4^+^ T cell metabolism from KBN mice treated with 2DG (Figure 6). A potential effect of metformin was supported by the fact that as for CD4^+^ T cells from lupus prone mice (3), both glycolysis and respiration were elevated in KBN CD4^+^ T cells. Contrary to lupus mice however (3), we did not observe an additional benefit from metformin in KBN mice, arguing that glucose is dominant in their pathogenesis. This result is consistent with highly glycolytic CD4^+^ T cells in human RA (39) in an hyper-reduced state (9). This is in contrast with lupus in which glycolysis plays an important role as evidenced by the effect of 2DG [(3), Choi et al. unpublished], but there is also evidence for a high level of oxidation in mice (8) and in patients (40, 41). Metformin has been shown to have a protective effect in collagen-induced arthritis (CIA), largely by preventing the expansion of Th17 cells and expanding the Treg population (42, 43). The effect of metformin on Tfh cells was not examined in these studies. In the KBN model, we did not observe an additional benefit of metformin over 2DG on the Th17 cells. Ongoing experiments with KBN mice treated with metformin alone should be informative whether the metabolic requirements of effector CD4^+^ T cells differ between the CIA and KBN models.

Metformin prevents the development of arthritis in the KBN serum transfer model, indicating a major effect on the innate immune effector cells (44). Interestingly, the addition of metformin to the 2DG treatment renders the autoantibodies produced by the KBN treated mice more pathogenic than those produced by mice treated with 2DG alone. This qualitative difference may due to their different ability to recruit innate immune cells based on their expression of Fc receptors specific to certain IgG isotypes. For instance, IgG1/GPI immune complex would preferentially recruit innate cells expressing FcγRIII (45), since in mice, IgG1 is the preferential ligand of FcγRIII, which is expressed on both neutrophils and monocytes (46). Anti-GPI IgG1 Abs are dominant in sera from KBN mice (16). However, FcγRIV, a preferential receptor for IgG2 isotypes also expressed on monocytes/macrophages and neutrophils, is required for KBN serum-mediated inflammation (47). Since the addition of metformin reduces the effect of 2DG on neutrophils, pDCs and CD11b-negative DCs, future studies should address the respective metabolic status of the innate subsets in the KBN model, as well as the relative distribution of IgG1 and IgG2 anti-GPI isotypes.

Overall, this study showed that inhibition of glycolysis significantly limits antibody-mediated pathology in the KBN model of RA, most likely through its effect on Tfh cells. The addition of metformin, which inhibits the compensatory switch to the oxidation of other substrates, limits the efficacy of glycolysis inhibition. Our results also suggest that a high glycolytic demand may be shared among Tfh cells in antibody-mediated autoimmune diseases.

## Author contributions

GA and S-CC designed, performed experiments, and co-wrote the manuscript, NK and LZ-S performed experiments, DR contributed reagents and participated to experimental design and data interpretation, LM conceived the study, participated to experimental design, data analysis and interpretation and wrote the manuscript.

### Conflict of interest statement

The authors declare that the research was conducted in the absence of any commercial or financial relationships that could be construed as a potential conflict of interest. The reviewer ET and handling Editor declared their shared affiliation.

## Abbreviations

2DG: 2-deoxyglusose
RA: rheumatoid arthritis
KBN: K/BxN
FLS: Fibroblast-like synoviocytes
Met: metformin
GPI: glucose-6-phosphate isomerase
Tg: transgenic
B6: C57BL/6J mice
ECAR: Extracellular acidification rate
OCR: Oxygen consumption rate
SrC: spare respiratory capacity
DC: dendritic cell
pDC: plasmacytoid dendritic cell
Tfh: CD44^+^CD279^+^CXCR5^+^Bcl6^+^Foxp3^−^CD4^+^ follicular helper T cells
Treg: CD25^+^Foxp3^+^CD4^+^ regulatory T cells
TEM: CD44^+^CD62L^−^ CD4^+^ effector memory T cells.
